# Visible Light Photocleavable Ruthenium-Based Molecular Gates to Reversibly Control Release from Mesoporous Silica Nanoparticles

**DOI:** 10.3390/nano10061030

**Published:** 2020-05-28

**Authors:** Yolanda Salinas, Oliver Brüggemann, Uwe Monkowius, Ian Teasdale

**Affiliations:** 1 Institute of Polymer Chemistry, Johannes Kepler University Linz, Altenberger Straße 69, 4040 Linz, Austria; oliver.brueggemann@jku.at (O.B.); ian.teasdale@jku.at (I.T.); 2 Linz School of Education, Johannes Kepler University Linz, Altenberger Straße 69, 4040 Linz, Austria; uwe.monkowius@jku.at

**Keywords:** mesoporous silica nanoparticles (MSNs), visible light photocleavage, molecular gates, reversibility, cargo release on demand, ruthenium complex

## Abstract

Herein we present hybrid mesoporous silica nanomaterials (MSN) with visible light-sensitive ruthenium complexes acting as gates. Two different [Ru(bpy)_2_L1L2]^2+^ complexes were investigated by grafting [Ru(bpy)_2_(4AMP)_2_](PF_6_)_2_ (RC1) and [Ru(bpy)_2_(PPh_3_)Cl]Cl (RC2) via two or one ligands onto the surface of mesoporous silica nanoparticles (MSNs), to give MSN1-RC1 and MSN2-RC2, respectively. The pores were previously loaded with a common dye, safranin O, and release studies were conducted. The number and position of the ligands were shown to influence the photocages behavior and thus the release of the cargo. Release studies from MSN1-RC1 in acetonitrile showed that in the dark the amount of dye released was minimal after 300 min, whereas a significant increase was measured upon visible light irradiation (ca. 90%). While successful as a photochemically-controlled gated system, RC1 was restricted to organic solvents since it required cleavage of two ligands in order to be cleaved from the surface, and in water only one is cleaved. Release studies from the second nanomaterial MSN2-RC2, where the complex RC2 was bound to the MSN via only one ligand, showed stability under darkness and in aqueous solution up to 180 min and, rapid release of the dye when irradiated with visible light. Furthermore, this system was demonstrated to be reversible, since, upon heating to 80 °C, the system could effectively re-close the pores and re-open it again upon visible light irradiation. This work, thus, demonstrates the potential reversible gate mechanism of the ruthenium-gated nanomaterials upon visible light irradiation, and could be envisioned as a future design of photochemically-driven drug delivery nanosystems or on/off switches for nanorelease systems.

## 1. Introduction

Mesoporous silica nanomaterials (MSNs) have been used for many years as outstanding candidates in the design of controlled-release nanocarriers because of their highly favored properties, such as thermal stability, biocompatibility, along with pore size tailoring and high cargo loading capabilities [1]. Moreover, their chemically active and large surface areas allow easy functionalization with different stimuli-responsive units [2]. Molecules can be attached to the surface as pore-blockers or “molecular gates” capable of controlling the access or transfer of mass from the inner pores upon the application of certain stimuli [3]. This closing–opening mechanism is a unique and special feature of these “gate-like” systems that have driven them, in the recent years, towards new designs in controlled-release drug delivery systems [4], development of selective optical chemosensing materials [2] and, very recently, for the preparation of responsive micromotors [5]. 

Among the physical stimuli investigated within molecular gates in MSNs, including temperature [6], pH [7], magnetic field [8], or their dual combination [9,10], the use of light has emerged as a very versatile tool that allows tunable excitation energy, remote control of the area and exposure time, and an appealing reversible on/off setup [11,12]. Accordingly, a wide range of photoresponsive chemistries have been developed [13]. In fact, a considerable number of reported MSN-based systems use UV light (at 250 nm) as a trigger of reversible photochemical processes to release the cargo, such as photodimerizations of the functionalized coumarin units [11], cis−trans photoconversions of azobenzene moieties [14], or also by photoisomerization of spiropyrans [15]. Moreover, the use of photocleavable linkers to control the opening/closing mesoporous systems has also been thoroughly explored with use of UV-photolabile groups, such as, for example, via cleavable 2-nitrobenzyl groups [16]. However, the main drawback of being opened upon UV light and closed under either visible light or thermally is a crucial concern in, for example, biomedical applications. Despite their excellent performance, such high energy irradiation is incompatible with biological media due to the low penetration and damage to biomolecules, hence a move to longer wavelength in the visible/NIR region is of critical importance [17]. 

Photosensitive ruthenium-based photocages can cleave one or sometimes two ligands in a photochemically-driven reaction [18], exchanging them for a solvent molecule, commonly H_2_O. The fundamental photoreaction is not only fast, it has high quantum yields in the visible region in a direct single-photon release mechanism. Depending on the ligand system, ruthenium complexes can be prepared in which the absorption bands are red-shifted through ligand modification [19], enabling photocleavage potentially at 600–700 nm [20]. If a ruthenium species is used which is of a benign nature [21], they can be potentially useful in biological systems. Indeed, while originally investigated for the release of biologically active molecules [22], such complexes have recently been used in materials science to prepare photocleavable hydrogels [23]. The reaction has also been demonstrated to be reversible according to the solvents and ligand combinations, leading to the development of dynamic self-healable photoresponsive polymers, reported by our group [24], and for reconfigurable surfaces developed by Wu and co-workers that can be photochemically manipulated in rapidly changing environments [25].

Indeed, the use of photocleavable ruthenium-based complexes as molecular gates in mesoporous hybrid systems has already been investigated. For example, Knežević and co-workers reported MSNs grafted with a [Ru(bipy)_2_(PPh_3_)]^2+^ complex coordinated via mercaptopropyl as a controlled release system of the dye sulforhodamine 101 [21], which is also used by the same group as anticancer treatment with amsacrine [26]. Stoddart, Sauvage, and co-workers also produced photochemically-induced releasing systems photocleavable ruthenium(II) polypyridine complex onto the surface of MSNs, but in this case through a grafted nitrile-containing derivative [27]. Herein, we prepared two different ruthenium complexes for functionalization of the surface of MSN nanoparticles. Firstly, the systems presented aimed at expanding the complexes applicable in this context, using pyridine ligands as the photoreleasing moieties and connecting via primary amines to allow simple grafting via urea chemistry. Furthermore, the second complex is designed to facilitate a reversible system and hence the ability to switch the release mechanism on and off. While Stoddart achieved a reversible on/off system with a combination of photocleavable ruthenium complexes and pseudorotaxanes, we demonstrate the on/off state in a much simpler system through temperature-driven complexation and, hence, closing of the pores.

## 2. Materials and Methods

### 2.1. Characterization Techniques 

Thermogravimetric analysis (TGA), transmission electron microscopy (TEM), dynamic light scattering (DLS), nuclear magnetic resonance spectroscopy (NMR), N_2_ adsorption–desorption analysis, and Fourier-transform infrared spectroscopy (FTIR) were employed to characterize the synthesized materials. TGA analyses were carried out on a Q5000, TA (TA instruments, New Castle, DE, USA) under nitrogen atmosphere (25 mL·min^−1^) in a platinum pan with a heating program from 40–900 °C at 10 °C·min^−1^. TEM images were obtained with a Jeol JEM-2200FS microscope (Jeol, Peabody, MA, USA). DLS measurements were taken on a Zetasizer Nano ZSP, from Malvern Instruments (Worcestershire, UK), in MilliQ water as dispersant (0.5 mg·mL^−1^) at 25 °C in disposable cuvettes DTS 0012 (from Malvern, Worcestershire, UK), previously sonicated (15 min) and filtrated (PTFE syringe filter, 0.45 μm). The MilliQ water used was obtained from a Millipore device with MilliPak express 400, 0.22 μm as a filter (from Millipore SAS, Molsheim, France). ^1^H and ^31^P NMR spectra were taken on Bruker Avance III 300 (Bruker BioSpin GmbH, Rheinstetten, Germany). The ^1^H NMR spectra were measured at 300 MHz using (CD_3_)_2_SO or CD_3_OD as deuterated solvents. The ^31^P NMR measurements were conducted at 121 MHz. N_2_ adsorption–desorption isotherms were recorded with a Micromeritics TriStar II 3020 surface area and porosimeter analyzer (Micromeritics, Norcross, GA, USA). The samples were degassed at 60 °C in vacuum for 2 h previous to measurement. The measurements were performed at 77.30 K and ca. 100 measurement points were recorded. Specific surface areas were calculated from the adsorption data within the low-pressure range using the Brunauer–Emmett–Teller (BET) model. Pore size was determined following the Barret–Joyner–Halenda (BJH) method. FTIR spectra were acquired with a Spectrum 100 FTIR spectrometer (PerkinElmer, Buckinghamshire, UK). The photoresponsive behavior of the ruthenium complexes was investigated by irradiation with a mercury-vapor (HBO) lamp (cut-off filter at 395 nm). Release studies were monitored by UV-Vis spectroscopy at each specific wavelength, done on a Lambda 35 UV-Visible spectrometer (PerkinElmer, Buckinghamshire, UK). 

### 2.2. Chemicals and Reagents

Safranin O (≥85%), tetraethyl orthosilicate (TEOS, 98%), 3-(triethoxysilyl)propylisocyanate (95%) and 4-(aminomethyl)pyridine (4AMP, 98%) were purchased from Sigma-Aldrich (Vienna, Austria). Cetyltrimethylammonium bromide (CTAB, >99%) was purchased from VWR (Vienna, Austria). Solvents were purchased from VWR and Alfa Aesar (Karlsruhe, Germany), and used as received, if not stated otherwise. [Ru(bpy)_2_(4AMP)_2_](PF_6_)_2_ (RC1) was synthetized according to the procedure described by our group [23,28], and [Ru(bpy)_2_(PPh_3_)Cl]Cl (RC2) was synthetized by following a reported procedure [29], see Figure S1.

### 2.3. Synthesis and Functionalization of Silica Mesoporous Nanoparticles (MSNs)

**Synthesis of MSN-0**. Mesoporous silica MCM-41 type nanoparticles were synthesized using a known reported procedure [30]. Surfactant CTAB (1.00 g, 2.74 mmol) was dissolved in 480 mL of 200 MilliQ water, followed by the addition of 3.5 mL of NaOH (2 mol·L^−1^). The temperature was then adjusted to 80 °C and TEOS (5.00 mL, 2.57 × 10^−2^ mol) was added dropwise to the surfactant mixture. The mixture was stirred for 2 h and a white precipitate was obtained. The precipitated solid was isolated by centrifugation and washed with deionized H_2_O until neutral pH. The sample was dried at 60 °C for 12 h. The surfactant was removed by calcination at 550 °C for 5 h and the final mesoporous silica nanomaterial MSN-0 was obtained.

**Synthesis of MSN1-NCO**. Calcined MSNs, MSN-0 (350 mg) and dye safranin O (98 mg, 0.28 mmol) were suspended in CH_3_CN (40 mL) in a round-bottom flask. To remove water from the pores of the calcined MSNs, 10 mL of the solvent were removed under nitrogen via azeotropic distillation. The suspension was stirred at room temperature for 24 h for loading the pores of MSN-0. To that suspension, 3-(triethoxysilyl)propyl isocyanate (350 μL, 1.41 mmol) was added and the reaction mixture stirred for 6 h at room temperature. The resulting solid (MSN1-NCO) which was already carrying the dye, was isolated by centrifugation, rinsed with 25 mL of acetonitrile, twice with 25 mL of distilled water in order to eliminate the possible residual dye, and finally dried under vacuum at 40 °C for 12 h.

**Figure 1:**
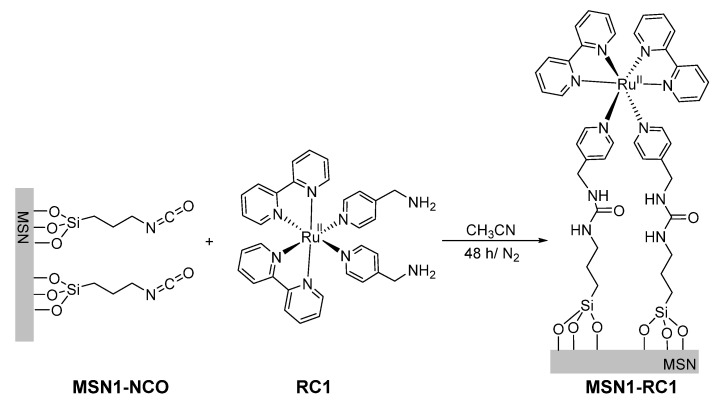
Preparation of MSN1-RC1 by grafting RC1 onto the mesoporous surface of MSN1-NCO.

**Synthesis of MSN1-RC1**. For the preparation of this material, 100 mg of MSN1-NCO and the complex RC1 (100 mg, 0.16 mmol) were suspended in CH_3_CN (10 mL) and Et_3_N (50 µL) in a round-bottom flask under nitrogen and in darkness (see Figure 1). The reaction mixture was stirred for 48 h at room temperature. The resulting solid functionalized with RC1 via urea moieties was isolated by centrifugation and washed with CH_3_CN (5 mL × 3) and finally ethanol (10 mL). The obtained product MSN1-RC1 was dried at room temperature for 12 h under darkness. 

**Synthesis of MSN2-Py**. For the urea formation, 220 mg of previous nanomaterial MSN1-NCO were suspended in dry CH_3_CN (35 mL) and under nitrogen, 4-(aminomethyl)pyridine (4AMP, 132 μL, 1.30 mmol) were added. The reaction mixture was stirred for 48 h at room temperature. The resulting solid, which is functionalized via urea moieties, was isolated and washed analogously to mesoporous silica nanomaterials MSN2-Py.

**Figure 2:**
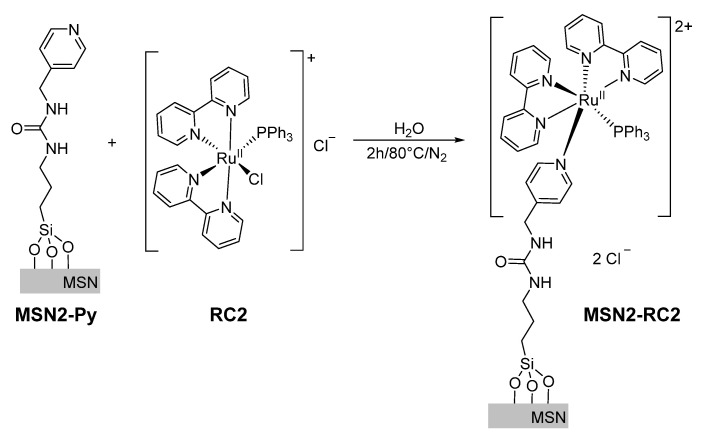
Preparation of MSN2-RC2 by grafting of RC2 onto the mesoporous surface of MSN2-Py.

**Synthesis of MSN2-RC2**. The previous loaded and functionalized solid MSN2-Py (75 mg) was suspended in H_2_O (5 mL). The synthetized ruthenium based complex RC2 (30 mg, 0.04 mmol) was dissolved in 5 mL of H_2_O and added to the suspension under inert conditions and exclusion of light. The reaction mixture was heated for 2 h at 80 °C (see Figure 2). The resulting material was then stirred for a total of 90 min and centrifuged and washed three times (30 min with 15 mL of water and 60 min with 15 mL of acetonitrile) at room temperature and exclusion of light until no more dye was observed at 520 nm (see absorption spectra in Supplementary Materials). The obtained product MSN2-RC2 was dried under vacuum for 12 h and stored in the dark.

### 2.4. Cargo Release Controlled under Visible Light Irradiation and Stability Studies 

In a typical release experiment, 5 mg of solid MSN1-RC1 or MSN2-RC2 were suspended in 12.5 mL of acetonitrile or in 12.5 mL of MilliQ water at pH 7, respectively, both under visible light irradiation (*λ* > 395 nm) or in the dark. Then, aliquots of 1.0 mL were collected at fixed times, filtered (to remove the silica nanoparticles), and the absorbance of safranin O at 520 nm was measured. 

Reversible partial dye release studies were followed only with MSN2-RC2 nanoparticles, as a function of temperature and irradiation time. For this experiment, 5 mg of MSN2-RC2 was suspended in 12.5 mL of MilliQ water at pH 7.4 and stirred for 30 min in the dark at room temperature, while aliquots of 1.0 mL were collected and replaced by fresh water, and the absorbance of safranin O at 520 nm was measured after specific times. After 30 min, the solid was irradiated with visible light triggering a rapid release of the entrapped dye. With the same samples the collecting and measuring procedure was followed for 90 min more. In a third step, the irradiation was stopped and the solid was stirred in the dark and heated up to 80 °C. After 60 min at those conditions, the solid suspension was cooled down to room temperature and the release was followed for 60 min. In the last step, the solid was irradiated again at room temperature and the release was followed as before for 120 min. The whole experiment was performed over 360 min. 

The stability of final nanomaterials was studied in PBS (7.4 and 5.2 related to biologically relevant media) and in MilliQ water at pH 7 and 5, by measuring the surface charge changes upon exposition to different media in the zeta potential of MSN1-RC1 and MSN2-RC2. 

## 3. Results and Discussion

### 3.1. Preparation of the Ruthenium(II) Gated-Mesoporous Silica Nanomaterials and Characterization

**Figure 3:**
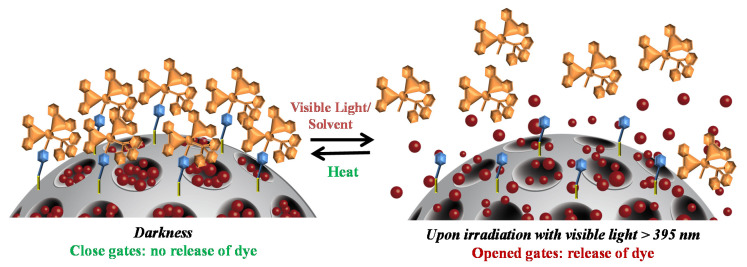
Scheme of the reversible light-triggered opening mechanism from loaded mesoporous silica nanoparticles surface functionalized with one of the proposed ruthenium-based complex systems (MSN2-RC2). Under visible light irradiation (>395 nm), the photochemical cleavage of the ligands leads to opened gates with a subsequent release of the dye in aqueous suspension. If heat is applied (at 80 °C), the system closes reversibly and no release is observed.

Our focus was the attachment of photosensitive ruthenium complexes of the form [Ru(bpy)_2_L1L2]^2+^ onto the silica surface which undergoes photosubstitution of their monodentate ligands by solvent molecules under irradiation with visible light. By using different ligands L1 and L2, one could control the time and solvent used to release a cargo molecule (dye), hence tailoring them for their application. Here, two different mesoporous silica nanomaterials were prepared capped with ruthenium-based complexes RC1 (two photocleavable ligands) and grafting [Ru(bpy)_2_(PPh_3_)Cl]Cl (called here RC2, once grafted containing one photocleavable ligand). A schematic representation of these novel hybrid nanomaterials and their light-responsive mechanisms is shown in Figure 3. Both nanomaterials were obtained by grafting the respective metal-complex via photocleavable, demobilized pyridine ligand onto the MSNs surface, which was previously loaded with a suitable dye, here safranin O (see MSN2-RC2 scheme in Figure 3, left). Upon irradiation with visible light, the photosubstitution of the pyridine ligands with solvent molecules removes the ruthenium-based caps and releases the dye from the pores (Figure 3, right). 

The mesoporous silica nanoparticles used here as a carrier support were prepared by a well-known sol-gel method [31], where the silica source TEOS polymerized under basic conditions at 80 °C around the hexagonal micellar arrangement of the surfactant CTAB, the pore-forming agent, in water. This cationic surfactant from the as-made nanomaterial was removed afterwards by calcination at 550 °C to obtain the nanomaterial MSN-0 containing empty pores. The mesopores of MSN-0 were filled up with the common dye safranin O, which allows an easy monitoring of its later release. The pores were then initially functionalized with 3-(triethoxysilyl)propyl isocyanate, which could form urea bonds either with 4-(aminomethyl)pyridine groups (material called MSN2-Py) or directly with RC1 (nanomaterial MSN1-RC1). The previously synthetized ruthenium complexes RC2 were attached in excess, with the pyridine moieties acting as ligands (from nanomaterial MSN2-Py) yielding MSN2-RC2. In order to ensure the efficient sealing of the pores, the final materials were extensively washed with water and acetonitrile to remove the excess of dye and unreacted ruthenium complexes. No more dye and complex were observed after three complete washing cycles, effectively followed by the absorbance of the dye at 520 nm.

**Figure 4:**
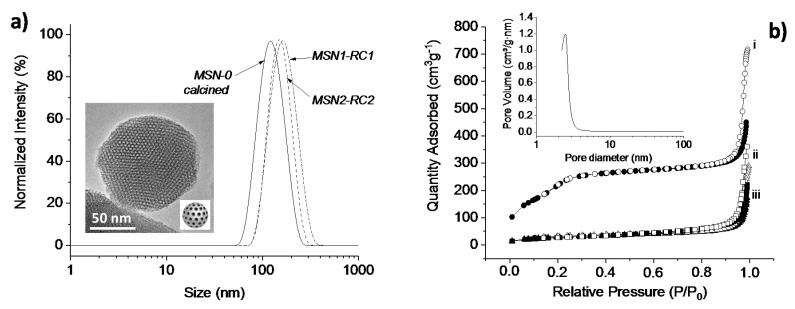
(**a**) Dynamic light scattering (DLS) measurements (hydrodynamic diameter, *H*_D_, via normalized intensity, collected data in Table 1) of MSN-0, and MSN1-RC1 and MSN2-RC2 (0.5 mg·mL^−1^ aqueous suspension); inset: TEM image of the calcined mesoporous silica nanoparticles MSN-0, where the typical hexagonal porosity is observed, scale bar: 50 nm; (**b**) nitrogen adsorption–desorption isotherms of the nanomaterials MSN-0 (**i**), MSN1-RC1(**ii**) and MSN2-RC2 (**iii**), full symbols and empty symbols stand for adsorption and desorption respectively. Inset: Barrett–Joyner–Halenda (BJH) pore size distribution of MSN-0.

The initial support nanomaterial MSN-0 was characterized by transmission electron microscopy and the typical hexagonal arrangement of mesopores was confirmed (see TEM image showed in Figure 4a inset). Spherical nanoparticles were detected with diameter ranging from 100–120 nm, values in agreement with the hydrodynamic diameters obtained by dynamic light scattering (see values in Table 1 and DLS in Figure 4a). The textural properties of these nanomaterials were determined by nitrogen adsorption–desorption analysis and as expected for this type of particles, a high total specific surface area of 869 m^2^·g^−1^ for the calcined MSNs was calculated using the BET model [32]. By applying the BJH model [33], the pore size distribution and the pore volume were determined. An average pore diameter of ca. 4 nm and pore volume of 0.67 cm^3^·g^−1^ were in the expected range for this type of MCM-41 nanoparticles. 

After loading the pores of MSN-0 with safranin O (suitable cargo molecule), the surface of the silica was functionalized with isocyanate (MSN1-NCO) or pyridine units (MSN2-Py). Both intermediate nanomaterials were characterized and compared with MSN-0. Visibly, no significant differences in their size were observed (*H*_D_ = 121 nm), while both showed a clear decreased surface area (163 and 218 m^2^·g^−1^) and pore volumes (0.31 and 0.37 cm^3^·g^−1^), respectively, which was attributed to the initial surface functionalization and to their highly loaded pores with the dye molecules, in consistency to those solids with filled mesopores reported in literature [30,34]. The presence of cylindrical pores was indicated by their isotherms, which typical shape type IV was obtained for the calcined and unloaded MSN-0, presenting a hysteresis shoulder between 0.1 and 0.3 of relative pressure (see Figure 4b and Figure S3). The hysteresis loop between 0.85 and 1.0 was indicative of a nonporous system, corresponding to the physical adsorption of the dye molecules inside the pores. In addition, a narrow distribution of the pore diameter was observed from the BJH pore size distribution curve (see inset in Figure 4b). In the following, the prepared ruthenium(II) complexes (RC1 and RC2) were attached to their intermediate solids (MSN1-NCO and MSN2-Py) by different pathways. The reactive amino group from RC1 formed a urea moiety with the isocyanate units immobilized already on the silica surface (MSN1-NCO) in acetonitrile while the second complex RC2 was attached directly by replacing the chlorine ligand from RC2 in water, leading finally to MSN1-RC1 and MSN2-RC2 nanomaterials, respectively. In order to corroborate the right reaction conditions, and thus the success of the urea formation, the model reaction between 4AMP and 3-(triethoxysilyl)propyl isocyanate was investigated and confirmed by ^1^H NMR (see Figure S2). The reactions involving the ruthenium(II) complex incorporation were kept in the dark in order to avoid any photocleavage during the reaction, which may lead to disrupt of their final light-responsiveness. After the characterization of final nanomaterials MSN1-RC1 and MSN2-RC2, even lower values were obtained for the surface area (in the range of 100 m^2^·g^−1^) and pore volumes (0.26 and 0.35 cm^3^·g^−1^), suggesting a potential incorporation of the ruthenium complexes to the surface and a probable blocking of the pores. This can also be interpreted by the very flat isotherms obtained for those nanomaterials (see Figure 4b, curves ii-iii) in comparison with the initial calcined MSN-0. It is worth noting that, after the different loading and functionalization steps, the pore size is expected to be not altered (data not shown in Table 1). 

The dye content and functionalities present in the nanomaterials were determined by thermogravimetric analysis (see Figure S4). The uptake capacity and entrapment efficiency of the dye were calculated by the following equations: uptake capacity (%) = 100 × (mass of cargo in MSNs)/(mass of cargo loaded MSNs) and uptake efficiency (%) = 100 × (mass or cargo loaded in MSNs)/(initial mass of cargo in feed). During the loading process, the amount of the dye taken up by the mesoporous silica nanoparticles was determined by subtracting the mass of the cargo in the supernatant from the total mass of the cargo in the initial solution. The uptake capacity and efficiency for the MSN1-NCO loaded with the dye safranin O was 6.9% and 46.7% respectively. These values were in agreement to similar systems [27]. 

Interestingly, similar weight loss values were obtained for both materials functionalized with the different ruthenium complexes (0.251 and 0.198 g of dye, and 0.093 and 0.080 g of ruthenium(II) complex per gram of silica for MSN1-RC1 and MSN2-RC2, respectively). The calcined mesoporous silica MSN-0 curve exhibited a weight loss below 100 °C commonly related to the loss of the water adsorbed. These values were in the same range of other gated systems developed for controlled release and constructed from mesoporous silica nanoparticles described in literature [35]. A narrow size distribution was obtained for MSN1-RC1 and MSN2-RC2 (PDI values collected in Table 1) nanoparticles. The hydrodynamic diameters of both final nanomaterials were higher than the intermediate functional materials MSN1-NCO and MSN2-Py (see values in Table 1 and DLS in Figure 4a), which may result from the final functionalization with the ruthenium (II) complexes.

**Figure 5:**
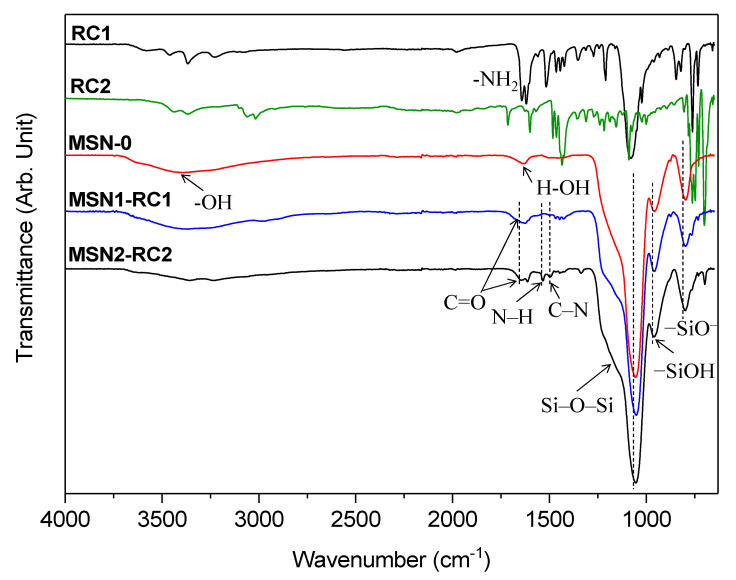
FTIR spectra of the ruthenium (II) complexes RC1 and RC2, of the calcined MSN-0, and of both final functionalized mesoporous silica nanomaterials MSN1-RC1 and MSN2-RC2, between 600 and 4000 cm^−1^: −SiO^–^ (795 cm^–1^), −SiOH (956 cm^–1^), −SiOSi− (1052 and 1231 cm^–1^), C−N (1495 cm^–1^), N−H (1530 cm^–1^), H−OH (1640 cm^–1^) C=O (1600-1700 cm^–1^), −NH_2_ (1615 cm^–1^), and –OH (3260 cm^–1^).

Additionally, Fourier-transform infrared (FTIR) characterization brought important insights to the chemical functionalization of the mesoporous silica-based nanoparticles (Figure 5). The FTIR spectrum of MSN-0 showed a characteristic large peak with a shoulder at 1052 and 1231 cm^−1^ corresponding to Si–O–Si, –SiOH at 956 cm^−1^, –SiO^–^ at 795 cm^−1^, assessed to the successful formation of the silica network. The broad peak at 3380 cm^-1^ was clearly corresponding to the hydroxyl groups (–OH) from the surface silanols (Si–O–H), while a peak at 1630 cm^−1^ was characteristic of H–OH water-twisting band of this type of inorganic silica support [36]. In the first functionalized material, the free reactive –NH_2_ signal at 1615 cm^−1^ of the 4-(aminomethyl)pyridine moieties from RC1 complex disappeared in MSN1-RC1, suggesting the urea formation with the complex, together with the presence of broad bands between 1615 and 1700 cm^−1^ assigned to C=O stretching vibrations from the urea signals. Likewise, the second functionalized material MSN2-RC2 showed peaks between 1612 and 1644 cm^-1^ which could be assigned to the urea C=O stretching signals, and signals at 1495 and 1530 cm^−1^ to C–N and N–H bending contributions, respectively, which agrees with reported similar materials [37]. Signals at 3240–3360 cm^−1^ were related to N–H stretching from the urea bond and the absence of a signal at 2260 cm^−1^ related to isocyanate stretching of –NCO, thus suggesting the successful functionalization of the complexes to the silica surface. The decrease of the –SiOH peak intensity also suggested that the functionalization occurred successfully. It is worth noting that, in both final materials, the Si–O–Si signals kept practically the same position and, hence, the silica network was stable during the loading and functionalization steps.

**Figure 6:**
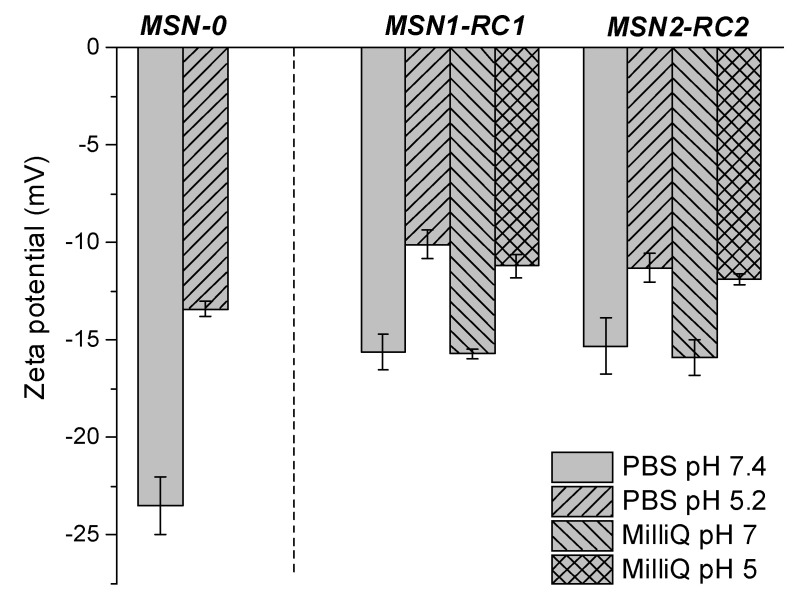
Zeta potential of the bare nanomaterial MSN-0 and of final ruthenium-functionalized nanomaterials MSN1-RC1 and MSN2-RC2 in both media MilliQ water and/or PBS at pH 7.4 and 5.2.

The stability of final nanomaterials MSN1-RC1 and MSN2-RC2 were tested and Zeta potential of MSN1-RC1 and MSN2-RC2 was measured in PBS (7.4 and 5.2 related to biologically relevant media) and in MilliQ water at similar pHs. No significant changes in the surface charge were observed within different media, around −16 mV at pH 7 and between −10 and −12 mV under slightly acidic conditions (see Figure 6 and Table S1). Therefore, these experiments supported the stability of the nanomaterials upon exposition to different media. Moreover, the surface charge of bare MSNs (MSN-0) was also measured in PBS at pH 7.4, and an expected highly negative zeta potential was obtained (−23.5 mV), due to the presence of negatively charged silanol groups on the surface; the value in the same order for the same type of particles reported in the literature under the same conditions [38] was a zeta potential of −22.4 mV (of MSNs in PBS at pH 7.4). Additionally, as expected for particles after functionalization (MSN1-RC1 and MSN2-RC2), the zeta potential becomes less negative due to the replacement of silanol groups with the corresponding ruthenium complexes.

### 3.2. Visible Light-Controlled Release by Close–Open Gate Nanomaterials 

**Figure 7:**
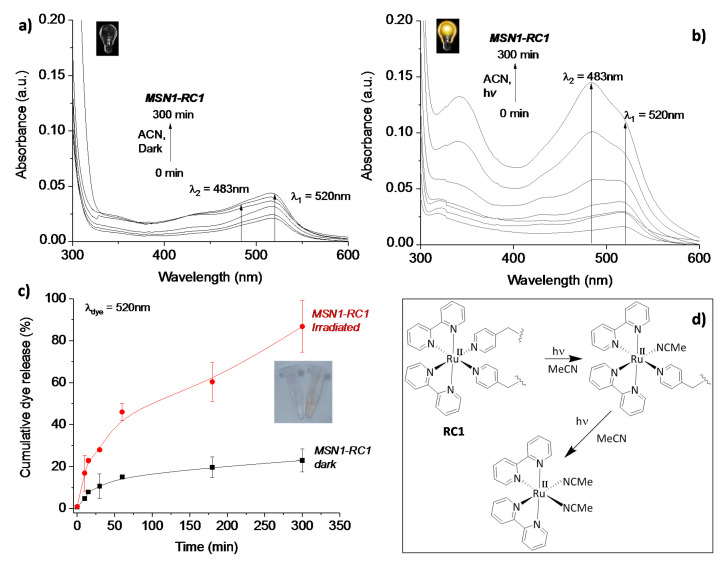
UV-Vis spectra from the release solutions measured from nanomaterial MSN1-RC1 under dark conditions (**a**) and after irradiation (**b**) with an HBO lamp (cut-off >395 nm, in acetonitrile) during 300 min; kinetic profiles of the released solutions of the safranin O at 520 nm before and after irradiation for 300 min (**c**); inset (**c**): Images of the solutions after 300 min of irradiation (**right**: pink-orange solution) or under dark conditions (**left**: colorless solution); schematic drawing of the photochemical cleavage of both pyridine ligands in acetonitrile which are linked to the silica surface of the nanoparticles (**d**).

Studies of light-driven dye release were carried out in the dark and under irradiation, monitoring the progress of dye released by UV-Vis spectroscopy. In order to perform the experiment, an HBO lamp with a cut-off filter >395 nm was located in front of the sample of MSN1-RC1 suspended in acetonitrile. The same experiments were carried out under dark conditions in acetonitrile and the release studies monitored for 5 h. As expected, it was observed that in the dark the amount of dye release (signal corresponding to safranin O was followed at 520 nm) was minimal after 300 min (<20% of dye was released, see Figure 7a), whereas a higher and significant increase in released dye was measured upon visible light irradiation (ca. 90%). Indeed, this could also be observed by the naked eye (see the released cargo in the inset images in Figure 7c). The modest dye release observed even under dark conditions could be related to possible instability of the material for prolonged treatment with acetonitrile, even in the dark (max. 300 min).

Of importance was also the signal detected at 483 nm, which was assigned to the cleaved ruthenium complex [Ru(bpy)_2_(NCMe)_2_]^2+^ released in the nanomaterial suspension at room temperature. These differences during irradiation measurements, shown in Figure 7a in the dark and in Figure 7b irradiated, were more evident after the sample was longer irradiated, up to 300 min, showing a clear increase of the peak at 483 nm in comparison to the one at 520 nm corresponding to the dye. Interestingly, the non-irradiated sample did not show any signal at 483 nm in the UV-Vis spectra (Figure 7a) even after 5 h. Hence, the potential light-triggered gate mechanism of the hybrid nanomaterial upon irradiation was demonstrated. Significantly, an increased signal detected under darkness after 300 min demonstrated the maximum stability of the system in the suspension (data not shown). In acetonitrile, the photocleavage follows a two-step process, as already reported [24], where the cleavage of the first ligand is significantly faster than the second one. In contrary, in water only one ligand is expected to cleave. 

**Figure 8:**
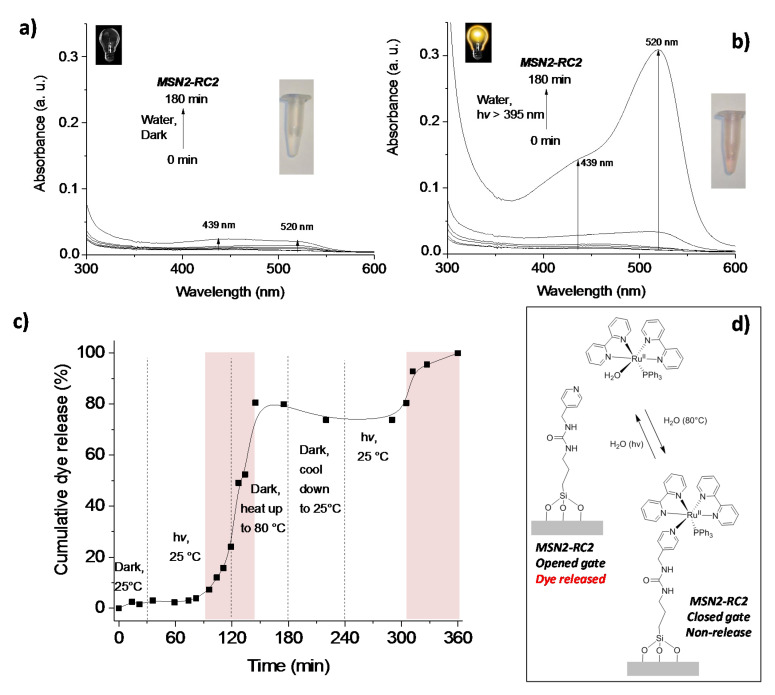
UV-Vis spectra of the dye solutions released from nanomaterial MSN2-RC2 under dark conditions (**a**) and after irradiation (**b**) with HBO lamp (cut-off >395 nm, in water) during 180 min; insets: Released solutions of the safranin O before (**a**) and after irradiation (**b**) for 180 min; cumulative partial release of dye (measured at 520 nm) from the nanomaterial as function of the irradiation and temperature changes (**c**) showing the reversible close–open behavior of the system and the areas marking when the release occurs; schematic drawing of the reversible photochemical cleavage of the pyridine ligand in water (**d**) releasing the dye due to opened gates after irradiation.

Etchenique and collaborators previously reported grafting of ruthenium phosphine complexes [Ru(bpy)_2_(PMe_3_)(APTES)] (with APTES = (3-aminopropyl)triethoxysilane) to a silica (glass) surface [18]. In their work, the authors demonstrated the successful and fast photocleavage of the ruthenium complex after irradiation with visible light, leaving behind the APTES ligand attached to the glass surface. Therefore, in order to prepare a favored pore-opening in aqueous media, a second material, MSN2-RC2, was designed to attach the metal complex RC2 by only one photocleavable pyridine ligand. In order to test its gate behavior, the release of dye was measured at 520 nm upon irradiation of the nanomaterial MSN2-RC2 in aqueous suspensions with visible light. Interestingly, it was noticed that a negligible release was observed within the first 60 min (<5%), however, rapidly started afterwards until ca. 75% of dye was released after 180 min (see Figure 8a,b). 

The release of the dye could also be observed clearly by naked eye (see inset solution image in Figure 8a,b). The dark experiment was performed for 300 min in total and it was observed that after 180 min the material started to release the dye. Moreover, the signal at 439 nm, indicative for the ruthenium complex, was detected in the suspension (see Figure S5, ESI). Therefore, the material showed stability in the dark and in aqueous solution only up to 180 min. In control experiments, it was additionally investigated whether the pyridine units close the pores to hinder the release under the same conditions in the dark in both water and acetonitrile. A complete release of the cargo from MSN2-Py was observed after 24 h (see Figure S6, ESI) demonstrating their non-blocking behavior. Additionally, the system MSN2-RC2 was studied in acetonitrile (see Figure S7, ESI). However, the same release studies demonstrated their non-blocking behavior even under dark conditions. 

After demonstrating the external light control of our hybrid nanosystem, the reversible character of the proposed closed–open gated mechanism of MSN2-RC2 was tested by carrying out few runs of dark–light–dark+heat–light (see Figure 8c). The cumulative release of the dye from the pores was measured at 520 nm and adjusted by varying the external conditions with time. Initially, after 30 min in the dark, the sample showed “zero cargo release” since the ruthenium complex blocked the pores (closed gate). After irradiating the sample for 90 min with visible light, an exponential release starts (releasing from <6% to 24% in 30 min) while continuing irradiation. After that, the irradiation was ceased and the sample was relocated under dark conditions and heated at 80 °C for 60 min, to allow the link to be formed again (see Figure 8d). Nevertheless, the dye released reached up to 80% before it stopped completely. Then, the suspension was kept under room temperature for 60 more minutes in the dark, observing no more dye released. Upon irradiation the release was observed to start again after 1 h, to increase 20% more during 2 h, tending to the saturation plateau. This study demonstrated successfully the potential reversible off-on behavior of the MSN2-RC2 nanosystem.

## 4. Conclusions

Mesoporous silica nanoparticles were functionalized with two different ruthenium complexes, linked to the outside pore surface via light cleavable bonds. Since the number and position of the ligands in the [Ru(bpy)_2_L1L2]^n+^ complexes are known to influence the photocages behavior, two different species were investigated, namely, [Ru(bpy)_2_(4AMP)_2_](PF_6_)_2_ (RC1) and [Ru(bpy)_2_(PPh_3_)Cl]Cl (RC2) and grafted onto the surface of mesoporous silica nanoparticles (MSNs) to give MSN1-RC1 and MSN2-RC2, respectively. The MSNs were loaded with the dye safranin O and, after careful characterization, release studies were conducted. From previous reports it was expected that RC1 could cleave both ligands in acetonitrile, but only one ligand in aqueous systems. Our investigations of MSN1-RC1 in acetonitrile showed that in the dark the amount of dye released was minimal after 300 min (<20% of dye was released), whereas a significant increase in released dye was measured upon visible light irradiation (ca. 90 %). While successful as a photochemically-controlled gated system, RC1 requires cleavage of two ligands in order to be cleaved from the surface, and it is known that only one of these is cleaved in H_2_O, hence this system is restricted to organic solvents. Therefore, we also investigated RC2, in which the ruthenium complex is bound to the MSN via only one ligand. Investigations of MSN2-RC2 showed stability under darkness and in aqueous solution up to 180 min, after which it appeared to become instable even in the dark. However, when irradiated with visible light, a rapid release of dye was observed. Furthermore, this system was demonstrated to be reversible, since, upon heating the system to 80 °C, the pores could be effectively re-closed. Reversibility could allow the nanosystem to be closed, due to the favoring of the reverse reaction and, hence, rebinding of the complex to the MSN surface. This process could be repeated, thus opening and closing the pores in response to the stimulus. This work, thus, demonstrates the potential reversible gate mechanism of the ruthenium-gated nanomaterials upon visible light irradiation and could be envisaged for future use in photochemically-driven drug delivery nanosystems or open–closed switches for nanorelease systems.

## Figures and Tables

**Table 1:** Characterization parameters from the mesoporous silica nanoparticles prepared before and after each functionalization step determined by nitrogen-adsorption–desorption isotherms and by dynamic light scattering (DLS). Surface areas (*S*_BET_) were determined by applying the Brunauer–Emmett–Teller (BET) model. Content of safranin O, functional linker, and corresponding ruthenium complexes (*α*_dye_, *α*_linker_ and *α*_RC_) were obtained by thermogravimetric analysis. Pore volumes (Pore vol.) and pore sizes were estimated by Barrett–Joyner–Halenda (BJH) model. Hydrodynamic diameters (*H*_D_) were obtained as average intensity values from at least three independent measurements.

Sample	*S*_BET_ m^2^/g	Pore vol._BJH_ cm^3^/g	Pore size_BJH_ nm	*H*_D_^[b]^ (PDI) nm	*α*_dye_ mmol/g_silica_	*α*_linker_ mmol/g_silica_	*α*_RC_ mmol/g_silica_
MSN-0	836	0.67	3.96	126 ± 33 (0.069)	-	-	-
MSN1-NCO	163	0.31	-	121 ± 28 (0.290)	0.478	1.070	-
MSN2-Py	218	0.37	-	121 ± 30 (0.146)	0.401	0.591	-
MSN1-RC1	105	0.26	-	193 ± 61 (0.309)	0.251	0.175	0.093
MSN2-RC2	100	0.35	-	187 ± 107 (0.205)	0.198	0.399	0.080

## Supplementary Materials

The following are available online at https://www.mdpi.com/2079-4991/10/6/1030/s1, Figure S1: Synthesis reactions of the ruthenium(II)-based complexes RC1 and RC2; Figure S2: Synthesis of the urea-containing silane compound in acetonitrile; Figure S3: Nitrogen adsorption–desorption isotherms of the nanomaterials (a) MSN-0, MSN1-NCO, MSN1-RC1 and (b) MSN-0, MSN2-Py and MSN2-RC2; full symbols and empty symbols stand for adsorption and desorption, respectively; Figure S4: Thermogravimetric analyses of the nanomaterials (a) MSN-0, MSN1-NCO, MSN1-RC1 and (b) MSN-0, MSN2-Py and MSN2-RC2; Figure S5: UV-Vis spectra of the release solutions measured from the nanomaterial MSN2-RC2 under dark conditions (a) and after irradiation (b) with HBO lamp (cut-off > 395 nm, in water) during 300 min; (c) filtrated release solutions from samples under dark conditions and after irradiation for 300 min; Figure S6: UV-Vis spectra of the release solutions measured from the nanomaterial MSN2-Py in (a) acetonitrile and (b) water during 1440 min under dark conditions; Figure S7: UV-Vis spectra of the release solutions measured from the nanomaterial MSN2-RC2 under dark conditions (a) and after irradiation (b) with HBO lamp (cut-off > 395 nm, in acetonitrile) during 300 min; Figure S8: UV-Vis spectra of the nanomaterial MSN2-RC2 washed for 90 min; Table S1: Zeta potential from the nanomaterial in PBS and MilliQ water at different pH conditions.
