# Supplementary material for: Visible Light Photocleavable Ruthenium-Based Molecular Gates to Reversibly Control Release from Mesoporous Silica Nanoparticles
Source: Nanomaterials (Basel). 2020 May 28;10(6):1030. doi: 10.3390/nano10061030 (PMC7352806; doi:10.3390/nano10061030)
Supplement: Supplementary file 1 [file nanomaterials-10-01030-s001.pdf]

Supporting Information

Visible light photocleavable ruthenium-based molecular gates to reversibly control release from mesoporous silica nanoparticles

Yolanda Salinas,[1,*] Oliver Brüggemann,[1] Uwe Monkowius[2] and Ian Teasdale[1]

1. Institute of Polymer Chemistry, Johannes Kepler University Linz, Altenberger Straße 69, 4040 Linz, Austria
2. Linz School of Education, Johannes Kepler University Linz, Altenberger Straße 69, 4040 Linz, Austria

*** Corresponding author:**

Email: yolanda.salinas@jku.at

Synthesis of the ruthenium(II)-based complexes RC1 and RC2:

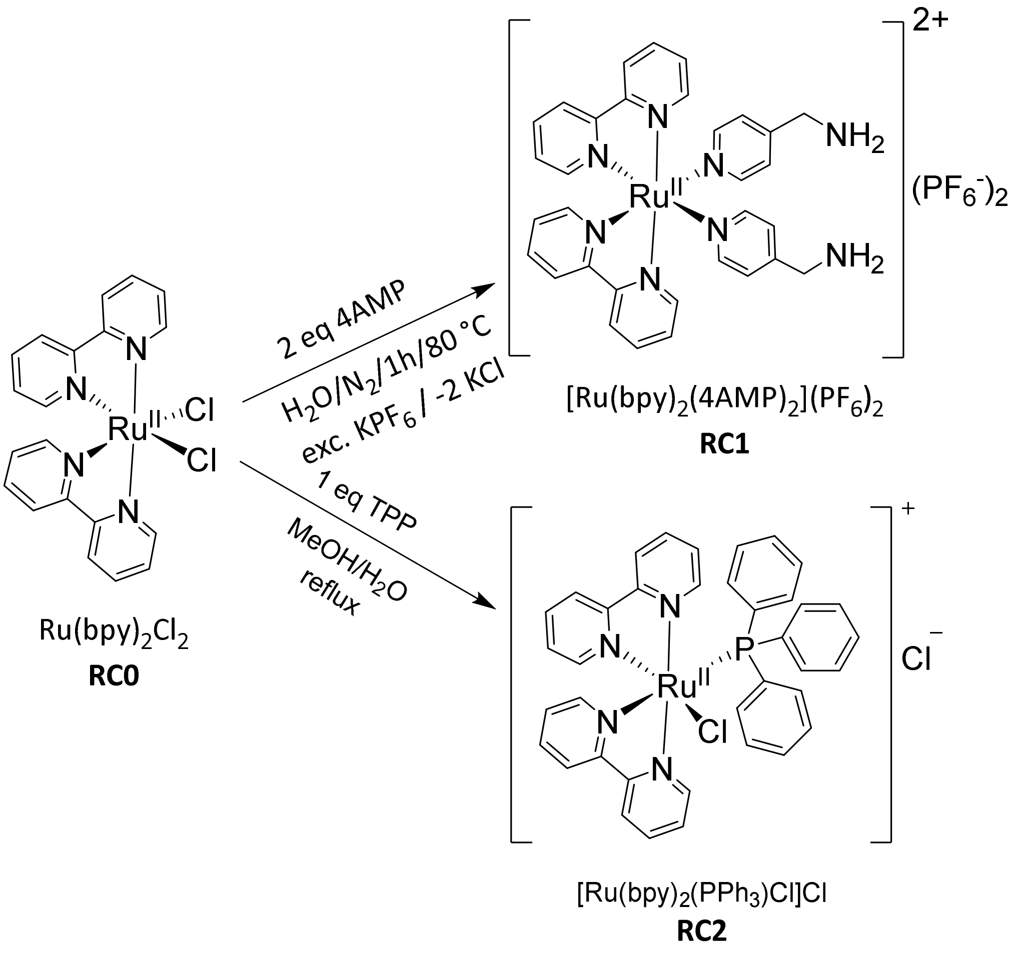

Figure S1. Synthesis reactions of the ruthenium (II) based complexes **RC1** and **RC2**.

Synthesis of [Ru(bpy)$_2$(4AMP)$_2$](PF$_6$)$_2$, RC1.

Ru(bpy)$_2$Cl$_2$ (**RC0**) was synthesized in analogy to the procedure described by our group.[1] Ru(bpy)$_2$Cl$_2$ (300 mg, 0.620 mmol) was suspended in 15 mL of water. The suspension was stirred at 80 °C under N_2 and after dissolution, 4-(aminomethyl)pyridine (4AMP, 350 μL, 3.45 mmol) was added. The reaction mixture was heated for 60 min. After cooling to room temperature the compound was precipitated with a saturated aqueous solution of KPF_6. The complex **RC1** was washed with water and dried obtaining an orange powder. (Yield: 76 %).

MS: m/z = 315.1 [Ru(bpy)$_2$(4AMP)$_2$]$^{2+}$ and 775.1 [Ru(bpy)$_2$(4AMP)$_2$](PF$_6$)$^{+}$.

^{1}H NMR (300.15 MHz, acetonitrile-d$_3$): δ 8.91 (d, J = 5.55 Hz, 2 H), 8.35 (d, J = 7.95 Hz, 2 H), 8.27 (d, J = 7.95 Hz, 2 H), 8.14 (m, 2 H and 4 H), 7.90 (m, 2 H and 2 H), 7.76 (t, J = 6.61 Hz, 2 H), 7.35 (t, J = 6.88 Hz, 2 H), 7.26 (d, J = 5.74 Hz, 4 Hc), 3.80 (s, 4 H), 1.49 (s, 4–NH$_2$).

^{13}C NMR (75.47 MHz, acetonitrile-d_3): δ 158.18, 158.13, 156.71, 153.39, 153.27, 152.90, 138.33, 138.00, 128.37, 124.80, 128.08, 124.46, 124.19, 44.44.

Synthesis of [Ru(bpy)$_2$(PPh$_3$)Cl]Cl, RC2.

[Ru(bpy)$_2$(PPh$_3$)Cl]Cl was synthesized according to a procedure described by L. Zayat *et al.*[2] Ru(bpy)$_2$Cl$_2$ (300 mg, 0.620 mmol) was dissolved in 10 mL of methanol and stirred for 15 minutes. Triphenylphosphane (TPP, 163 mg, 0.622 mmol) was added and the mixture was stirred for another 15 minutes (see Figure S1). After complete dissolution, 3 mL of water were added and the mixture was refluxed for 2 hours. The solvent was removed by rotary evaporation and then resuspended in 10 mL of acetone. After keeping the reaction mixture for 1 h at 0 °C, the complex **RC2** was filtered and dried in the desiccator. (Yield: 73 %).

^1H NMR (300.15 MHz, methanol-d_4): δ 9.24 (d, J = 4.79 Hz, arom. 2H), 8.64 (d, J = 8.39 Hz, arom. 1H), 8.58 (d, J = 8.39 Hz, arom. 1H), 8.48 (d, J = 7.75 Hz, arom. 1H), 8.37 (d, J = 7.75 Hz, arom. 1H), 8.14 (t, J = 8.39 Hz, arom. 1H), 8.00 (t, J = 7.95 Hz, arom. 2H), 7.76 (t, J = 7.75 Hz, arom. 1H), 7.37 (m, arom. 12H), 7.22 (m, arom. 8H), 6.86 (t, J = 7.10 Hz, arom. 1H).

^{31}P NMR (121 MHz, methanol-d_4): δ 42.31.

Synthesis of 1-(pyridin-4-ylmethyl)-3-(3-(triethoxysilyl)propyl)urea

Under nitrogen, 4AMP (0.50 mL, 2.0 mmol), 3-(triethoxysilyl)propyl isocyanate (0.35 mL, 1.4 mmol) and Et_3N (30 µL) were added to a round-bottom flask containing CH_3CN (30 mL) (see reaction scheme in Figure S2). The reaction mixture was stirred for 12 h at room temperature. The solvent was removed under vacuum in the rotary evaporator yielding stoichiometrically a yellowish-like oil.
^1H NMR (300.15 MHz, methanol-d_4): δ 8.45 (d, J = 7.4 Hz, arom. 2H), 7.36 (d, J = 7.4 Hz, arom. 2H), 6.61 (s, NH_2), 6.24 (s, NH_2), 4.38 (s, 2H), 3.83 (q, J = 5.7 Hz, 6H), 3.15 (t, J = 4.5 Hz, 2H), 1.61 (m, 2H), 1.23 (t, J = 5.7 Hz, 9H), 0.64 (t, J = 9.3 Hz, 2H).

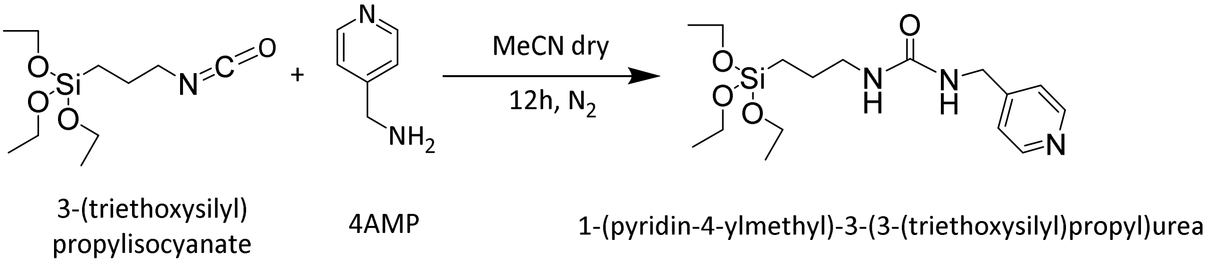

Figure S2. Synthesis of the urea-containing silane compound in acetonitrile

Characterization of the nanoparticles:

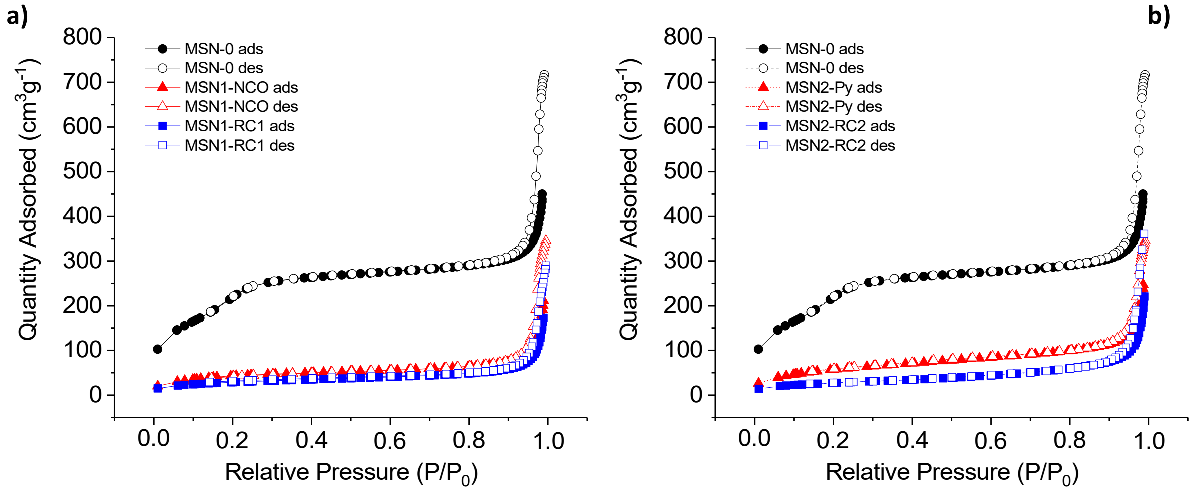

Figure S3. Nitrogen adsorption-desorption isotherms of the nanomaterials a) MSN-0, MSN1-NCO, MSN1-RC1 and b) MSN-0, MSN2-Py and MSN2-RC2; full symbols and empty symbols stand for adsorption and desorption, respectively.

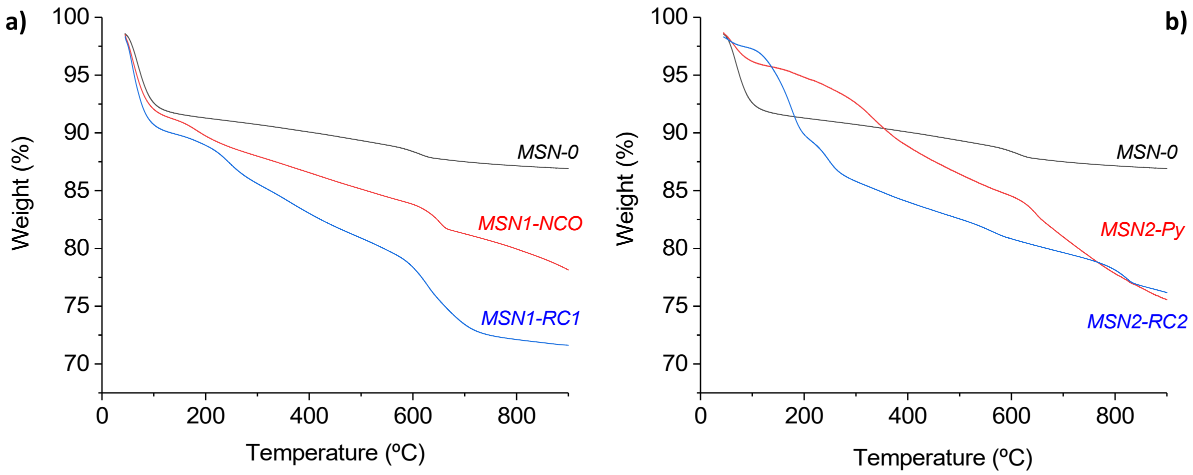

Figure S4. Thermogravimetric analyses of the nanomaterials a) MSN-0, MSN1-NCO, MSN1-RC1 and b) MSN-0, MSN2-Py and MSN2-RC2.

Table S1. Zeta potential from the nanomaterial in PBS and MilliQ water at different pHs.

Nanomaterials	**Zeta potential (mV) PBS pH 7.4**	**Zeta potential (mV) PBS pH 5.2**	**Zeta potential (mV) MilliQ pH 7**	**Zeta potential (mV) MilliQ pH 5**
MSN-0	-23.5± 1.50	-13.4± 0.40	-	-
MSN1-RC1	-15.6± 0.93	-10.1± 0.74	-15.7± 0.23	-11.2± 0.60
MSN2-RC2	-15.3± 1.45	-11.3± 0.72	-15.9± 0.90	-11.9± 0.27

Dye release studies:

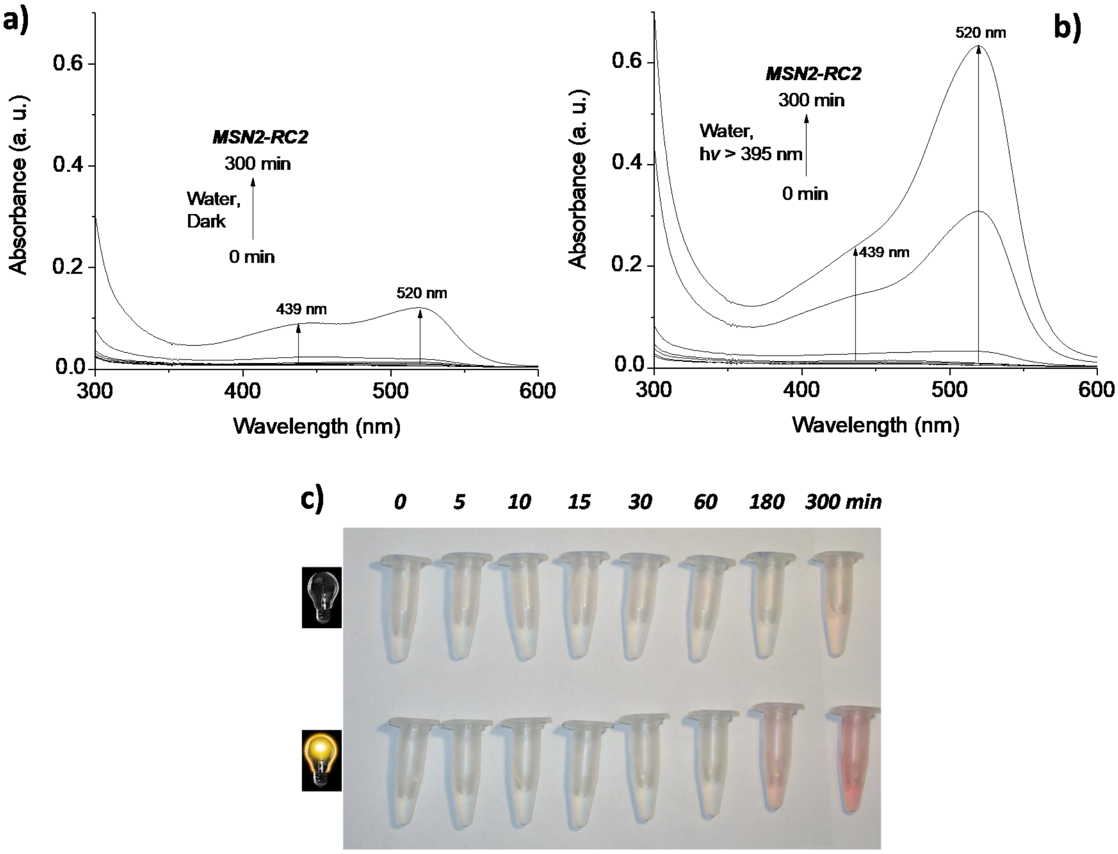

Figure S5. UV-Vis spectra of the release solutions measured from the nanomaterial MSN2-RC2 under dark conditions (a) and after irradiation (b) with HBO lamp (cut-off > 395 nm, in water) during 300 min; (c) filtrated release solutions from samples under dark conditions and after irradiation for 300 min.

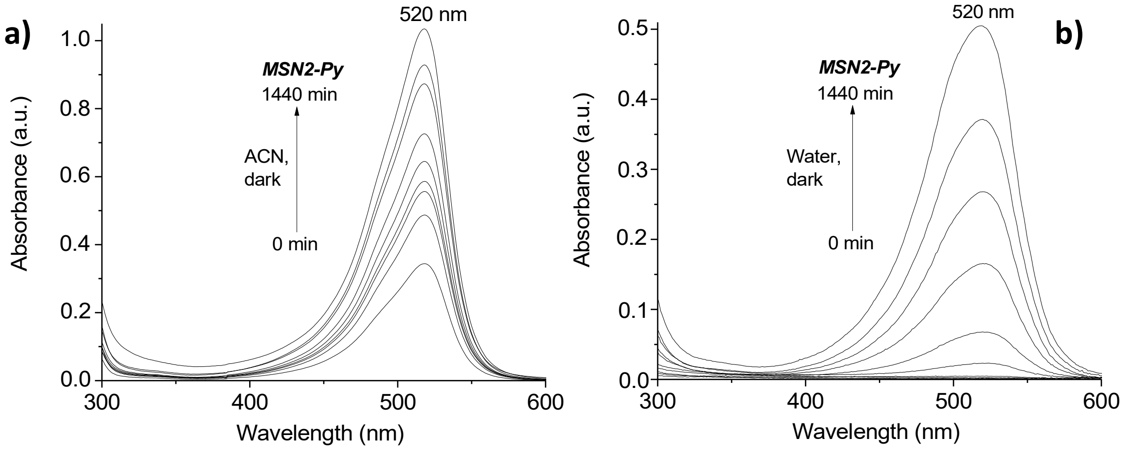

Figure S6. UV-Vis spectra of the release solutions measured from the nanomaterial MSN2-Py in (a) acetonitrile (a) and (b) water during 1440 min under dark conditions.

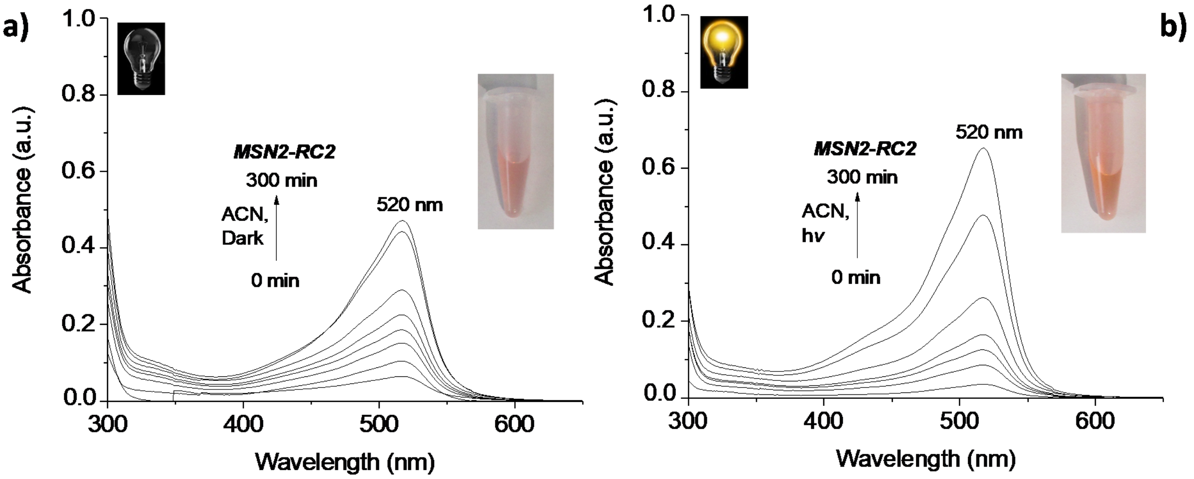

Figure S7. UV-Vis spectra of the release solutions measured from the nanomaterial MSN2-RC2 under dark conditions (a) and after irradiation (b) with HBO lamp (cut-off > 395 nm, in acetonitrile) during 300 min.

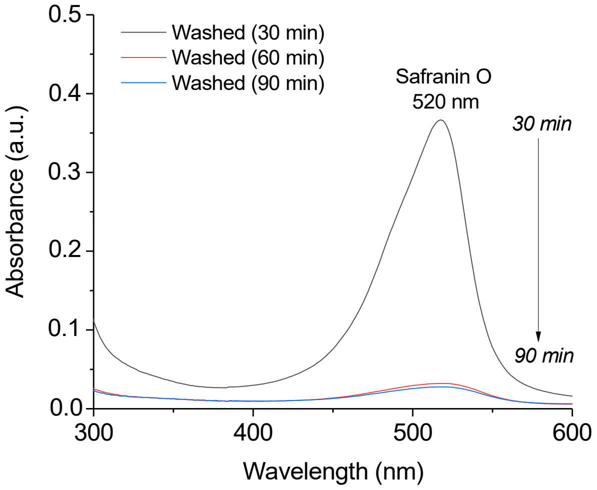

Figure S8. UV-Vis spectra of the nanomaterial MSN2-RC2 washed for 90 min.

References

[1] S. Theis, A. Iturmendi, C. Gorsche, M. Orthofer, M. Lunzer, S. Baudis, A. Ovsianikov, R. Liska, U. Monkowius, I. Teasdale, Metallo-Supramolecular Gels that are Photocleavable with Visible and Near-Infrared Irradiation, Angewandte Chemie International Edition, 56 (2017) 15857-15860.

[2] L. Zayat, M. G. Noval, J. Campi, C. I. Calero, D. J. Calvo, R. Etchenique, A New Inorganic Photolabile Protecting Group for Highly Efficient Visible Light GABA Uncaging, ChemBioChem, 8 (2007) 2035-2038.
